# Immune Mechanisms of Plaque Instability

**DOI:** 10.3389/fcvm.2021.797046

**Published:** 2022-01-11

**Authors:** Teresa Gerhardt, Arash Haghikia, Philip Stapmanns, David Manuel Leistner

**Affiliations:** ^1^Charité – Universitätsmedizin Berlin, Department of Cardiology, Berlin, Germany; ^2^DZHK (German Centre for Cardiovascular Research), Partner Site Berlin, Berlin, Germany; ^3^Berlin Institute of Health at Charité – Universitätsmedizin Berlin, Berlin, Germany

**Keywords:** plaque erosion, trained immunity, clonal hematopoiesis, infection, COVID-19, stress, gut dysbiosis, atherosclerosis

## Abstract

Inflammation crucially drives atherosclerosis from disease initiation to the emergence of clinical complications. Targeting pivotal inflammatory pathways without compromising the host defense could compliment therapy with lipid-lowering agents, anti-hypertensive treatment, and lifestyle interventions to address the substantial residual cardiovascular risk that remains beyond classical risk factor control. Detailed understanding of the intricate immune mechanisms that propel plaque instability and disruption is indispensable for the development of novel therapeutic concepts. In this review, we provide an overview on the role of key immune cells in plaque inception and progression, and discuss recently identified maladaptive immune phenomena that contribute to plaque destabilization, including epigenetically programmed trained immunity in myeloid cells, pathogenic conversion of autoreactive regulatory T-cells and expansion of altered leukocytes due to clonal hematopoiesis. From a more global perspective, the article discusses how systemic crises such as acute mental stress or infection abruptly raise plaque vulnerability and summarizes recent advances in understanding the increased cardiovascular risk associated with COVID-19 disease. Stepping outside the box, we highlight the role of gut dysbiosis in atherosclerosis progression and plaque vulnerability. The emerging differential role of the immune system in plaque rupture and plaque erosion as well as the limitations of animal models in studying plaque disruption are reviewed.

## Clinical Relevance

Atherosclerotic plaques are characterized by hyperlipidemia and non-resolving inflammation, tightly linked by complex innate and adaptive immune processes (1, 2). Atherosclerosis is the underlying pathology of cardiovascular disease (CVD). Instability of atherosclerotic plaque and subsequent atherothrombosis are the most common causes of myocardial infarction (MI) (3). The 17.8 million deaths attributed to CVD in 2017 (4) mark it to being a persisting pre-eminent global health problem despite highly effective options available to control conventional cardiovascular risk factors (4, 5). Immunity and inflammation likely contribute substantially to this residual risk (5, 6). We currently witness an exciting series of trials that highlight immune pathways as a central target for cardiovascular secondary prevention and clinically affirm the inflammation-hypothesis of atherosclerosis.

Recently, the CANTOS trial substantiated an immunomodulatory therapy to improve cardiovascular outcomes. In this study, targeted inhibition of the inflammatory cytokine Interleukin-1β by canakinumab, a neutralizing antibody, markedly reduced adverse cardiovascular events as compared to placebo in a large past-MI study cohort with “residual inflammatory risk (RIR)” as defined by a high sensitivity C-reactive protein level >2 mg/L (7). Another clinical outcome study, the COLCOT trial, investigated a second preventive treatment strategy after recent MI using the broad anti-inflammatory agent colchicine or placebo. The outcome showed a 23% relative reduction in adverse cardiovascular events after a median follow up of 22.6 months in the treatment group, mainly driven by reductions in the rates of stroke and angina requiring revascularization (8). A similarly meaningful effect was recently confirmed for patients with chronic coronary disease in the LoDoCo2 trial demonstrating significant cardiovascular risk-reduction upon Colchicine treatment (HR 0.69 at 0.5 mg daily) (9), although this was not associated with a difference in all-cause mortality (10). IL-1 strongly induces Interleukin-6 (IL-6) (11) and has been attributed a causal role in human coronary heart disease (12). Current trials have shown beneficial effects of the receptor antagonists Tocilizumab and Ziltivekimab on myocardial salvage (13), biomarkers of inflammation in MI patients (13, 14), or patients at high atherothrombotic risk (15). However, a concomitant increase in triglyceride levels associated with tocilizumab has prevented its use in large clinical trials (11). The investigators of the recently published “CRP apheresis in Acute Myocardial Infarction (CAMI-1)” study moved downstream of IL-6 in the inflammatory cascade, directly targeting the inflammatory marker high-sensitivity C-reactive protein (hsCRP) (16). In this non-randomized pilot study, 66 patients with acute ST-elevation-MI were treated with repeated CRP-aphereses, reducing the mean hsCRP concentration by 53.0 ± 15.2%. On the one hand, the study demonstrates a correlation between the systemic CRP level, the extent of myocardial damage and the restriction of myocardial function. On the other hand, it introduces CRP-apheresis as an interesting and safe, but technically challenging method to target direct detrimental effects of CRP after acute MI. However, the study was insufficiently powered for outcome analyses. Thus, the therapeutic effect will need to be analyzed in a larger, controlled, randomized trial (16).

Beyond the IL1-IL6-CRP axis and colchicine, a plethora of other promising targets within the innate and adaptive arm of the immune system are being explored (11, 17, 18). While these landmark trials have launched a thrilling new era of treatment in CVD, a more precise and conclusive understanding of the underlying immune mechanisms is essential to routinely apply and personalize the new therapeutic concepts. Recent novel findings based on new techniques have expanded and refined our knowledge of atherosclerotic plaque instability. In the following, we integrate conventional paradigms with new discoveries, to showcase today's understanding how maladaptive immune responses drive plaque instability. We discuss different manifestations of instability, plaque rupture and plaque erosion, and summarize the accumulating evidence that they have little in common but their clinical appearance. We aim to provide a more global perspective by considering systemic influences that lead to acute plaque destabilization and highlight the limitations that animal models entail in the endeavor to explain how stable plaques become unstable.

## Atherosclerotic Plaque Formation and Features of Plaque Instability

Preferably at sites of low endothelial shear stress, increased blood levels of low-density lipoprotein (LDL) and other cardiovascular risk factors, such as metabolic syndrome or cigarette smoking (19), favor loss of endothelial integrity and endothelial activation. This allows for accumulation of lipids in the arterial intima, where they are oxidized, aggregate and are engulfed by smooth muscle cells (SMCs) and macrophages, causing foam cell formation (20–23). Modified lipids act as chronic stimuli for innate and adaptive immune-responses that orchestrate a smoldering low-grade inflammation of the vessel wall (18). Subsequent cell apoptosis and necroptosis, complicated by failed efferocytosis (dead cell removal by phagocytes), cause formation of a lipid-rich necrotic core (NC) and production of thrombogenic tissue factor (21). NC components and inflammatory cells lead to degradation of plaque-stabilizing extracellular fibrous matrix (ECM) like collagen and proteoglycans and thinning of the fibrous cap (20). Hypoxia-inducible factors produced by cells contained in the NC, promote pathologic neoangiogenesis, which favors intraplaque hemorrhage and further expansion of the NC. Unresolved inflammation triggers plaque calcification, which further reduces mechanical stability of the plaque (20). Thus, features of plaque instability include large NCs (>24–50% of total lesion area), high amounts of inflammatory cells, thin fibrous caps (23 ± 19 μm), reduced ECM, abundant neovascularization and intraplaque hemorrhage and calcification (24).

### Plaque Rupture and Plaque Erosion—The Concept of Plaque Instability Needs Revision

Instability of coronary atherosclerotic plaque culminates in abrupt vascular thrombus formation that impedes blood flow and leads to critical myocardial ischemia (25). Clinically, this often manifests as life-threatening Acute Coronary Syndromes (ACS) (4). Experts long equated coronary thrombosis with rupture of the typical “vulnerable plaque,” the thin-capped fibroatheroma (TCFA) (26). Recent technological and conceptual breakthroughs have initiated a paradigm shift and diversified understanding of underlying pathomechanisms. Contemporary data from intravascular ultrasonographic imaging studies provided evidence that the traditional “vulnerable plaque” phenotype with TCFA does not correlate with the likelihood of clinical destabilization (6). This led to abandoning the classic “vulnerable plaque” concept (27, 28).

Besides the well-studied plaque rupture, *in vivo* high-resolution (10–15 μm) intravascular plaque imaging by optical coherence tomography (OCT) identified superficial erosion with thrombus formation on an intact fibrous cap in 25–40% of ACS patients (29–34). Likely due to better control of traditional risk factors, this percentage seems to be ever increasing (35, 36) and superficial erosion may become the dominating plaque morphology in the foreseeable future. While discussing mechanisms of plaque instability, we must keep this novel dichotomy in mind, since, as discussed below, underlying pathomechanisms seem to differ substantially and could well lead to a fundamental shift in the management of ACS patients sooner rather than later. Other, less frequent substrates for coronary thrombosis are calcified nodules in 2–8% (29, 30, 32) and dissection of the vessel wall in 1–2% (30, 31) of ACS events. In this review, we focus on the two predominant mechanisms of ACS, plaque rupture and plaque erosion.

## Mouse Models of Plaque Instability

Monogenetic knock out mice for the low-density lipoprotein (LDL) receptor (*Ldlr*^−/−^)- or Apolipoprotein E (*Apoe*^−/−^) are the two most widely used animal models of atherosclerosis (37). Both are suboptimal to study coronary plaque instability. First, atherosclerosis develops rarely in the coronary arteries, but rather in the aorta and other larger vessels (38) in which hemodynamics and vascular histology are naturally very different. Second, plaque disruption and thrombotic occlusion almost never occur spontaneously, which has in part been attributed to lower surface tension and smaller vessel diameter in mice than in humans (39). Double-KO (dKO) mice for the high-density lipoprotein receptor gene (scavenger receptor b1; *Scrb1*^−/−^) and *Apoe* or *Ldlr* develop in many aspects human-like CA-lesions and spontaneous plaque disruption with occlusive coronary arterial atherothrombosis, but exhibit complex comorbidities and die prematurely at early age (40). *Apoe*^−/−^ or *Ldlr*^−/−^ dKO mice that carry an additional heterozygous function impairing mutation in the Fibrillin-1 gene (*Fbn1*^*C*1039*G*+/−^) show features of plaque vulnerability including increased apoptosis of smooth muscle cells (SMCs), larger necrotic cores, increased macrophage- and T-cell infiltration, intraplaque hemorrhage and hypervascularization after 10 (41), 12/24 (42) or 35 (43) weeks of feeding with atherogenic high-fat diet. Remarkably, in one study, A*poe*^−/−^ *Fbn1*^*C*1039*G*+/−^ mice had atherosclerosis also in the coronary arteries, frequently suffered strokes (64% of cases) and 70% died suddenly (43). However, rates of spontaneous plaque rupture varied greatly between studies, occurring in 5% (41) to 70% (43) of A*poe*^−/−^ *Fbn1*^*C*1039*G*+/−^– and 20% of *Ldlr*^−/−^ *Fbn1*^*C*1039*G*+/−^(42) mice and mice showed no evidence of plaque erosion.

Besides genetic KO models, some surgical models have been tested. Tandem stenosis of the carotid artery of hypercholesterolemic *Apoe*^−/−^ mice induced features of instability, including intraplaque hemorrhage (50%) and fibrous cap disruption (32%) 7 weeks postoperatively. Notably, no eroded plaque was identified (44). Based on studies that show endothelial denudation and flow disturbance as likely pathogenic triggers in plaque erosion (45, 46), electrical injury of the carotid artery adventitia of *Apoe*^−/−^ mice, followed by flow perturbation by constrictive periadventitial cuff after an intermediate healing-period was used as a model to specifically study mechanisms that pertain to plaque erosion (47), but naturally, this cannot be used as a causal model for plaque erosion. In a promising recent study, pressure overload in hearts of chow diet fed *Apoe*^−/−^ mice by minimally invasive transverse aortic constriction surgery induced coronary lesions in 93% of animals, most frequently in the LAD (54%). Histology identified MI in 74% of animals, in 34% of cases due to plaque rupture and in 13% due to formation of a thrombus on an intact fibrous cap, i.e. plaque erosion (48).

In sum, mouse models of acute plaque instability are scarce and, in most cases, unreliable or rather artificial. Experimental modeling of the rupture/erosion dichotomy has been impossible until recently, although it is urgently needed. A few novel surgical approaches that have recently shown to induce plaques that rupture or erode could soon yield seminal results.

## Immune Cells Crucially Drive Atherosclerosis

Plaque stability fundamentally depends on the level of inflammatory cell infiltration (49). Multidimensional single-cell RNA sequencing and mass cytometry of mouse and human atherosclerotic plaque have provided insights into the cellular landscape of atherosclerotic plaque at unprecedented granularity (50–55), comprehensively reviewed in Hill et al. (56). Although the relative frequency of immune cell populations varies between studies, macrophages and αβ T-cells consistently represent the most abundant cell types and overwhelming evidence supports their crucial role in atherosclerosis development and progression. We concentrate mainly on these two and neutrophils in the following, although a plethora of different innate and adaptive cells are relevant to atherosclerosis progression. For an in depth discussion of the role of dendritic cells, mast cells, B cells, natural killer (NK) cells, and unconventional T-cells in atherosclerosis, we refer the reader to other recent and up-to-date review articles, respectively. Briefly, dendritic cells accumulate in plaques and form foam cells, regulate T-cell activation and proliferation by antigen-presentation, mediate efferocytosis and secrete immune-modulating cytokines and chemokines (18, 57, 58). Mast cells accumulate in the arterial adventitia and are activated to degranulate, releasing various mediators, some of which destabilize the plaque (proteases, proinflammatory cytokines), but also others that promote plaque stability by inhibiting thrombus formation or providing oxygen to hypoxic areas of the plaque (59, 60). B-cells produce antibodies and may have proatherogenic or disease-limiting properties, in both, an antibody-mediated as well as an antibody-independent manner, depending on their sub-phenotype and antibody profile. Their complex role in atherogenesis and –progression is reviewed in Roy et al. (18) and Sage et al. (61). NK cells seem to play a subordinate role in atherosclerosis: although NK-cell derived perforin and granzyme B were demonstrated to aggravate disease (62), a later study using a selective NK-cell loss-of-function model did not confirm a direct effect of these cells on lesion size or -stability (63). Similarly, depletion of γδ T-cells, an MHC-independent subset of T-cells that carry TCRs composed of γ- and δ-chains, had no effect on the development of atherosclerosis in high-fat diet fed TCRδ^−/−^ApoE^−/−^ mice (18, 64). NK T-cells are found in minor amounts in atherosclerosis and promote NC-growth and inflammation by production of cytotoxic and proinflammatory cytokines and chemokines (18, 65, 66).

### Macrophages and Neutrophils

**Figure 1:**
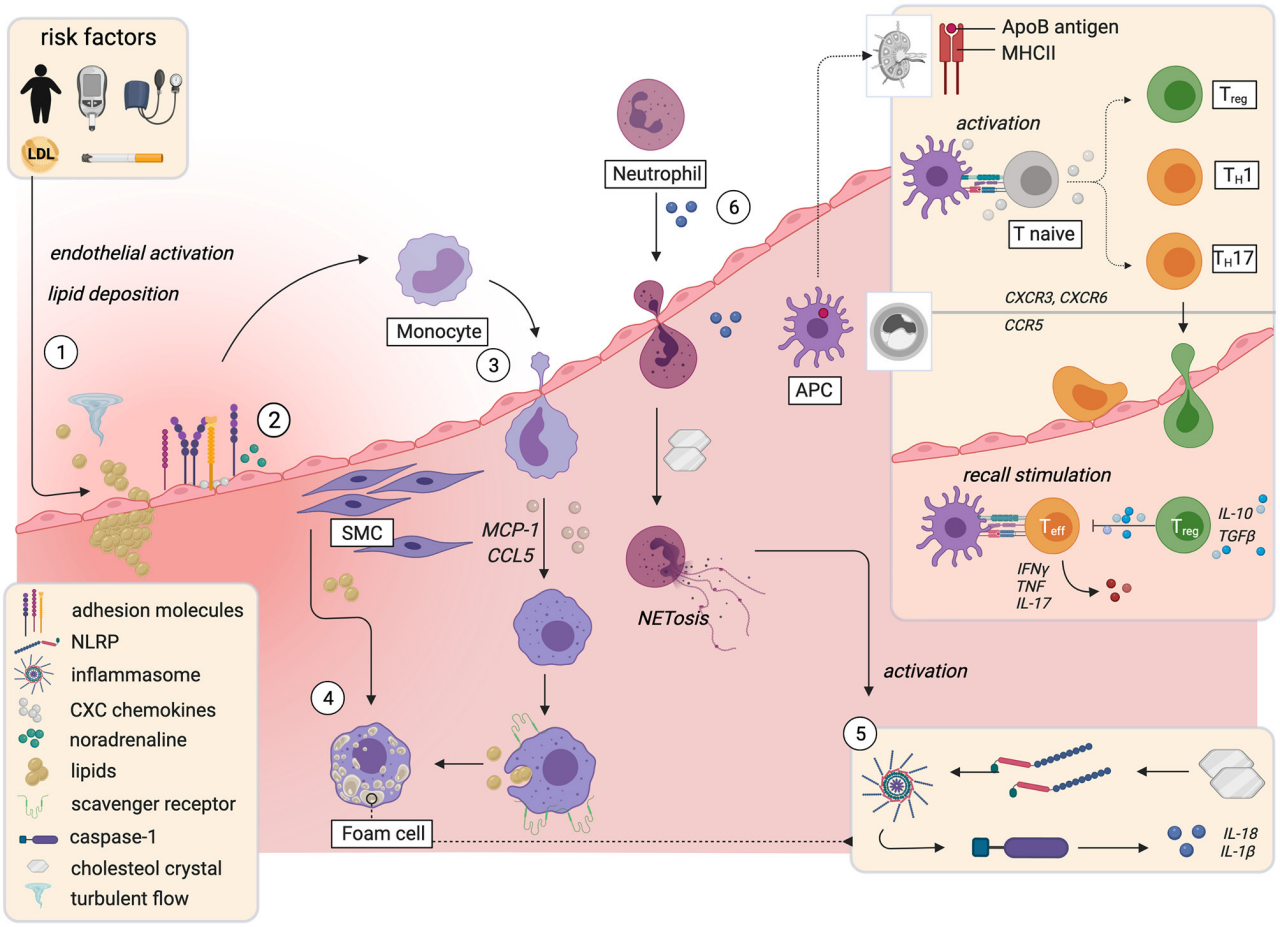
Immune mechanisms implicated in initiation and progression of atherosclerosis. The influence of risk factors and turbulent flow conditions activate the endothelium and prime the endothelial sites for focal retention of apolipoprotein B-containing lipoproteins (1). This causes upregulation of leukocyte adhesion molecules and the presentation of chemokines and cytokines on the luminal surface, partially mediated by noradrenaline (2). Monocytes extravasate into the plaque and differentiate into macrophages that take up lipids and form foam cells (3). Smooth muscle cells acquire macrophage characteristics and also become foam cells (4). Cholesterol crystals that accumulate in foam cells prime formation of the NLRP3-inflammasome, which activates caspase-1. Caspase-1 triggers activation and release of IL-1β and IL-18 (5). The resulting proinflammatory milieu leads to neutrophil recruitment and NETosis, which further enhances the release of proinflammatory mediators (6). In lymph nodes, T-cells recognize fragments of apolipoproteinsB100 and other plaque antigens. Upon recall stimulation in the plaque, they differentiate into proinflammatory effector- or anti-inflammatory regulatory sub-phenotypes (box upper right corner). APC, antigen presenting cell; ApoB, apolipoproteinB100; LDL, low density lipoprotein; MHCII, major histocompatibility complex II; NET, neutrophil endothelial trap; NLRP; NACHT, LRR and PYD domains-containing protein 3; SMC, smooth muscle cell; T_eff_, T-effector cell; T_H_, T-helper cell; T_reg_, regulatory T-cell. Created with BioRender.com.

Atherosclerosis is initiated by risk factor- and flow-induced endothelial damage, which predisposes for focal retention of apolipoprotein B (apoB)-containing lipoproteins in the arterial intima. It was long thought to be a predominantly lipid-driven disease (67). Despite the indisputable causal role of lipid deposition, it is the ensuing smoldering inflammation and maladaptive immune responses that propel plaque progression and link traditional risk factors with atherosclerosis (1) (Figure 1). Smoking, hyperglycemia, and hypertension activate vascular smooth muscle cells (SMCs) and the vascular endothelium, especially at sites of disturbed, non-laminar flow (11, 68). This entails upregulation of adhesion molecules like ICAM-1, ICAM-2, VCAM-1, E-Selectin, and P-Selectin and the presentation of tethered CXC chemokines and cytokines on the luminal surface of the endothelium (2, 69, 70), in part mediated by increased activity of the sympathetic nervous system through locally released noradrenaline (70). Monocytes traffic to the site of injury, extravasate, and differentiate into macrophages, a process mainly dependent on monocyte chemoattractant protein-1 (MCP-1) and CC-Chemokin-Ligand-5 (CCL5) (11). Monocyte-derived macrophages overexpress scavenger receptors like CD36, LOX-1, SR-A and CXCL16 (71) and excessively engulf LDL, giving rise to dysfunctional lipid-laden foam cells (72) that accumulate cytoplasmic cholesterol crystals (73).

A self-amplifying vicious circle perpetuates the inflammatory response: in plaque macrophages, cholesterol crystals act as danger signals that prime formation and activation of the NACHT, LRR and PYD domains-containing protein 3 (NLRP3)-containing inflammasome (73), a key signaling platform that activates caspase-1, which mediates proteolytic activation of the cytokines IL-1β and IL-18 (73). IL-1β up-regulates a plethora of proinflammatory cytokines, induces monocyte differentiation into macrophages (74), and triggers an endothelial cell response that further stimulates myeloid recruitment (1, 75) and leukocyte diapedesis and differentiation (76). Additionally, IL-1β-induced chemokines and activated SMCs recruit granulocytes to the plaque (74, 77, 78). Neutrophil exposure to cholesterol crystals induces formation of neutrophil extracellular traps (NETs) (77), extracellular networks of decondensed chromatin, histones, and antimicrobials that are part of the neutrophil host defense against pathogens (79). NETs act as crucial priming cue for proinflammatory cytokine production in macrophages through activation of the NLRP3 inflammasome. *AopE*^−/−^ mice that lack key enzymes required for NETosis consistently displayed smaller lesions (77).

In sum, lipid-deposition and endothelial activation and dysfunction entail the accumulation of myeloid cells in the vascular wall. Potentiated by neutrophils, this creates a proinflammatory cytokine environment and gives rise to foam cells.

Of note, arterial macrophages do not follow the classical M1/M2 subset framework (80), but are very heterogeneous in polarization and function (51, 81, 82). Not all of them drive atherosclerosis progression and some even promote healing and resolution of inflammation (18). Macrophage heterogeneity increases with disease progression (82). Recent single-cell experiments have, at a transcriptional level, identified at least five distinct macrophage-subsets in murine atherosclerotic aortas (82), of which at least four (marked with ^*^) have also been identified in human plaque (81): resident^*^, inflammatory^*^, interferon-inducible cell (IFNIC)^*^, aortic intima-resident (Mac^AIR^) and Trem2^+^ (triggering receptor expressed on myeloid cells-2) foamy macrophages^*^. Resident macrophages, the only macrophage population in healthy arteries, populate the adventitia and have been suggested to limit arterial stiffness by inhibition of collagen production by SMCs (83). If they promote or alleviate atherosclerosis, is unknown (18). They mainly derive from embryonic CX3CR1^+^ precursors, and, especially in advanced plaques, are maintained through local proliferation (84). Inflammatory macrophages and IFNICs, which both differentiate from circulating monocytes and are only present during atherosclerosis, are clearly proinflammatory and drive lesion progression (18). Inflammatory macrophages highly express inflammatory chemokines and show increased expression of inflammasome components and proinflammatory cytokines such as IL-6, IL-1β and TNF (50, 51), while IFNICs produce proinflammatory interferon-I (51, 53). Another macrophage subset that drives lesion progression is the intima-resident Mac^AIR^ subset: in mouse models of atherosclerosis, Mac^AIR^ depletion reduced lipid deposition and fatty streak formation (85). This specialized resident macrophage subset differentiates from infiltrating monocytes shortly after birth, but, during inflammation progression may also be replenished from circulating monocytes (18, 85). Macrophage-derived Trem2^+^ foam cells, on the other hand, have been shown to suppress inflammatory gene expression and alleviate vascular inflammation (53, 86).

### αβ T-Cells

The profound inflammatory myeloid response in atherosclerosis is accompanied by infiltration of adaptive immune cells, which, mainly through cytokines and antibodies, decisively regulate plaque inflammation (87) (Figure 1). Activated T-lymphocytes are present in all stages of atherosclerosis (88). CD4^+^ T cells comprise 25–38% of leukocytes in mouse and human plaques (1, 50–52). T cells home to the aorta in a manner partially dependent on the surface expressed chemokine receptors CCR5 (89), CXCR3 (90), and CXCR6 (91, 92). Single cell data shows that plaque T cells have gene signatures associated with activation, exhaustion, inflammation, and cytotoxicity (52). T cell responses in atherosclerosis are antigen specific and major histocompatibility complex (MHC)-dependent and -restricted (49, 87, 93, 94). Of note, the best-known atherosclerosis-antigens are peptides from apoB100, the core protein of LDL and its oxidized form oxLDL (93–95). Consequently, atherosclerosis involves a relevant autoimmune response (1, 87, 96), although it is not a classical autoimmune disease, initiated by detrimental immunity against itself (87).

Antigen-loaded dendritic cells (DCs) that have migrated to lymph nodes, typically initiate T-cell activation in atherosclerosis (97), while plaque-resident DCs and -macrophages as well as adventitial B cells display antigen for recall stimuli that induce antigen-experienced memory T-cells (1, 49, 98). Upon specific recognition of cognate MHC/antigen complex by the T cell receptor (TCR) and co-stimulatory molecules, T-cells respond by clonal expansion and differentiation. CD4^+^ T-cells further differentiate into distinct regulatory or effector sublineages, namely T-helper 1 (T_H_1),−2 (T_H_2),−9 (T_H_9),−17 (T_H_17),−22 (T_H_22), follicular helper T (T_FH_)-cells, or T_reg_ subtypes (Type 1 regulatory (Tr1) or *Forkhead Box P3* (*FOXP*3)^+^ T_reg_ cells) (87, 98). Each subset displays a distinct transcriptional program and cytokine pattern that may promote or curb atherosclerosis (98). Cytokines and costimulatory signals from antigen presenting cells determine the microenvironment that polarizes T-cells toward immunogenic or tolerogenic responses (58).

The balance between pro-inflammatory effector T-cells (T_eff_) and anti-inflammatory regulatory T-cells (T_reg_) determines the fate of the plaque (98, 99). Briefly, T-helper 1 (T_H_1) cells are proatherogenic (1, 100, 101) and dominate mouse and human plaques (52). They express proinflammatory interferon-γ (IFNγ), interleukin (IL)-2 and-3 and tumor necrosis factor (TNF), which activate macrophages and reduce plaque stability (1). Activation of the NLRP3 inflammasome in macrophages by cholesterol crystals (73) is linked to T-helper 1 (T_H_1) differentiation and IFNγ production via caspase-1 dependent proteolytic activation of the co-stimulatory cytokine IL-18 (102). Similarly, most MHC-I–dependent cytotoxic CD8^+^ T cells, which secrete perforin, granzyme B, TNF, and IFNγ, and preferentially accumulate in the fibrous cap (103), drive plaque instability by enhancing inflammation and promoting necrotic core formation. In contrast, *FOXP*3^+^T_reg_ cells are atheroprotective and sustain immune system homeostasis, balancing out proatherogenic responses (104–106). They secrete inhibitory cytokines such as IL-10 (107) and plaque-stabilizing transforming growth factor (TGF)-β (108), induce anti-inflammatory macrophages (109), and regulate proliferation of proinflammatory effector cells (106, 109). T_H_17 function in atherosclerosis remains controversial. T_H_17 cells produce IL-17A and -F, both of which induce pro-inflammatory cytokines (87), and IL-17A blockade reduces experimental atherosclerosis (110). However, a T_H_17 subtype induced by IL-6 and TGFβ produced atheroprotective IL-10 (107, 111) together with IL-17 (112). Some studies further attribute plaque-stabilizing function to T_H_17 cells (113). Similarly, the function of the quantitatively less important T_H_2, T_H_9, T_H_22, and T_FH_ subsets in atherosclerosis remains a matter of debate (1, 98).

## Maladaptive Immune Responses that Destabilize the Plaque

The crosstalk between innate and adaptive responses in the atherosclerotic plaque creates a delicate balance between pro- and anti-inflammatory mediators (18). Some recently discovered harmful immune mechanisms may help us understand why proinflammatory factors eventually prevail, causing plaque disruption and dramatic clinical sequelae.

### Trained Immunity

Memory was long seen as an exclusive hallmark of the epitope-specific adaptive arm of the immune system. Recent findings teach us otherwise. Epigenetic changes are long-term alterations in gene expression through modification of DNA accessibility, e.g. through acetylation or methylation, without permanent genetic changes (114). KDM5 (lysine demethyltransferase 5) and Set7 (SET domain containing 7, histone lysine methyltransferase) are among important epigenetic enzymes (115). Exogenous insults such as the fungal ligand β-glucan or lipopolysaccharide (LPS) from microbes (116, 117), but also endogenous “sterile danger signals” (118) such as modified LDL and other atherogenic stimuli may induce sustained epigenetic, metabolic and functional reprogramming in myeloid cells, natural killer cells and innate lymphoid cells that enhance their cytokine production upon restimulation, even with a stimulus different from the initial “training-cue” (115, 116). This adaptive-like, but antigen-independent hyperresponsiveness of innate immune cells to recall stimulus is known as “trained immunity” (119). It is important to note that trained immunity does not refer to one particular regulatory program, but to different long-lasting (114), but reversible, epigenetic alterations of transcriptional pathways induced by different stimuli (120). Although beneficial in the setting of recurrent infection, trained immunity can lead to a maladaptive hyperinflammatory immune response in chronic inflammatory diseases like atherosclerosis (114, 120).

**Figure 2:**
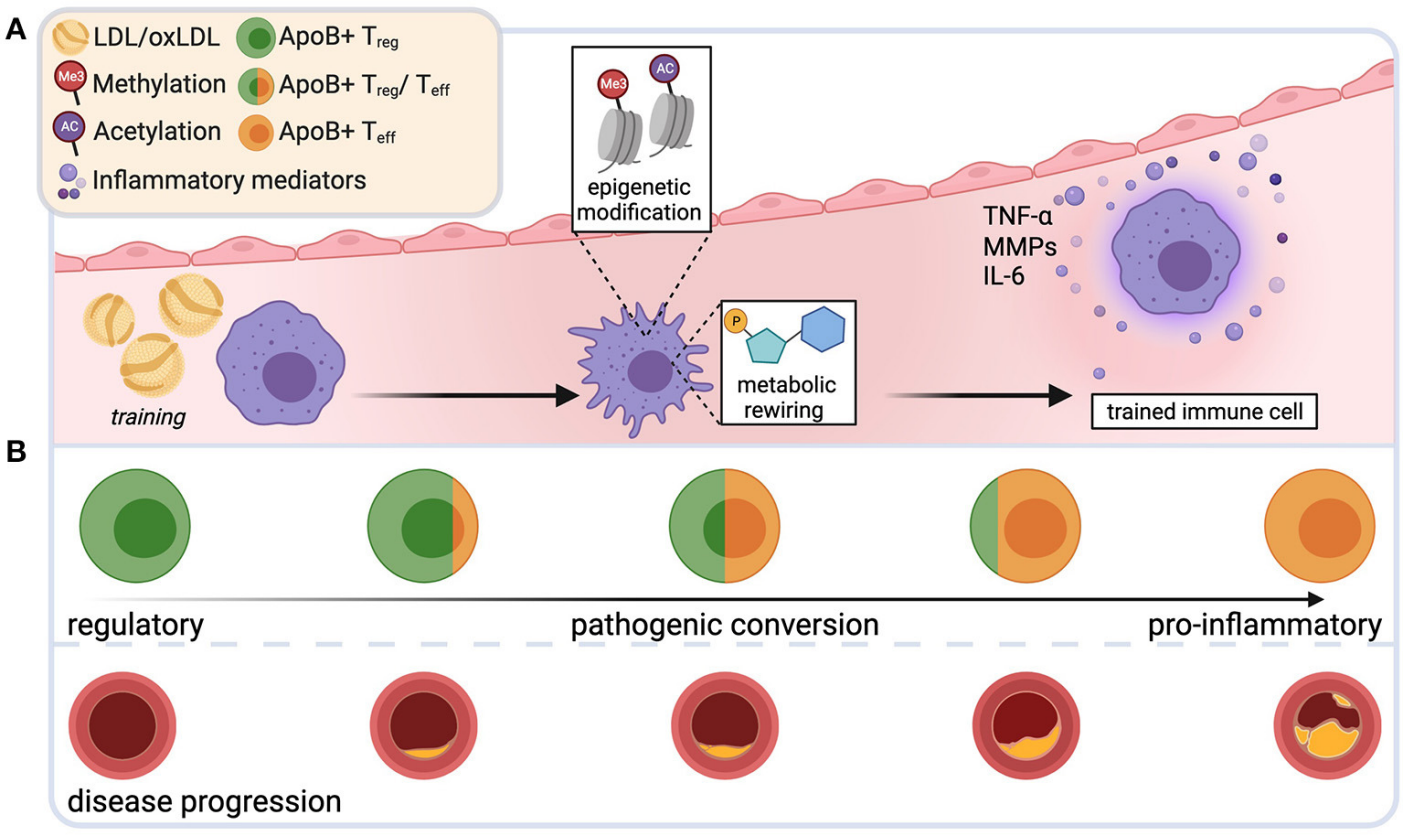
Novel immune mechanisms that drive plaque instability. Repetitive exposure to low-density lipoprotein (LDL) and its oxidized form (oxLDL) during plaque progression may induce epigenetic changes and metabolic rewiring in innate immune cells. Such trained cells produce more proinflammatory mediators upon restimulation, aggravating the proinflammatory bias in the plaque milieu **(A)**. During early atherosclerosis, apolipoproteinB100-specific T-cells are mostly regulatory T cells (T_reg_). During disease progression, these cells undergo a phenotypic switch and acquire phenotypic and functional characteristics of harmful effector T cells (T_eff_) **(B)**. Created with BioRender.com.

Compared to naïve controls, macrophages primed with modified LDL (“training”) retained more intracellular cholesterol and released more TNF-α, IL-6 and matrix metalloproteinases (MMPs), Zn^2+^-based destabilizing catalytic proteases that degrade extracellular matrix (ECM) (121), upon proinflammatory restimulation *in vitro* (23) (Figure 2A). This was based on epigenetic modification, since the “training effect” was prevented by the blockade of histone methyltransferases (23). A concomitant metabolic rewiring involved mTOR/HIF1α-dependent upregulation of glycolytic capacity and oxidative phosphorylation (122). LDL-trained macrophages (23) and monocytes from patients with familial hypercholesterolemia (123) showed enrichment of the transcriptionally permissive chromatin mark histone H3 trimethylated at lysine 4 (H3K4me3) in the promotor region of genes that encode proatherogenic and proinflammatory cytokines. In line with this, blood monocytes from patients with severe coronary artery atherosclerosis exhibit characteristics of a trained phenotype in terms of histone hypermethylation, cytokine production, and metabolic reprogramming (124, 125). Given the short half-life of blood monocytes, it is noteworthy that hypercholesterolemia-induced changes in the epigenome may occur not only in mature myeloid cells, but also at the level of myelopoiesis: high-fat-diet (HFD)-induced (=“training”) hypercholesterolemia in *Ldlr*^−/−^ mice led to profound proinflammatory epigenetic reprogramming of long-lived bone marrow granulocyte-macrophage progenitor cells and a hyperinflammatory response of their progeny that persisted for several weeks after discontinuation of the HFD despite normalized cholesterol levels and systemic inflammatory markers (118). This was mediated by the NLRP3-inflammasome and IL-1β (118).

Although deeper mechanistic and clinical evidence of the direct detrimental effect of trained immunity in atherosclerosis is needed, it is tempting to speculate that the observed accelerated myelopoiesis, hyperresponsiveness, and increased production of proinflammatory mediators in response to repetitive stimulation with modified plaque lipids may promote plaque inflammation that ultimately fuel plaque instability and disruption. Given that innate immune memory is a relatively new discovery in the context of atherosclerosis, essential questions remain unanswered. Does the training effect and duration vary between the different innate immune cell types? Does it directly influence the players of the adaptive immune system that are omnipresent in the plaque? Is trained immunity thoroughly reversible and can we therapeutically exploit this? Could some of the “genetic” cardiovascular risk be due to training-induced epigenetic alterations being passed on in the germline?

### Phenotypic Instability of LDL-Induced Protective Autoimmune Cells

As outlined above, T_regs_ are clearly atheroprotective, stabilize the plaque, and outbalance proinflammatory immunity. In atherosclerotic *Ldlr*^−/−^ mice, hypercholesterolemia was shown to initially drive T_reg_ differentiation and homing to the inflamed aorta (126). T_reg_ numbers significantly correlated with LDL plasma levels in subclinical human atherosclerosis (127). This was likely antigen-driven, since hypercholesterolemia increased T-cell receptor (TCR) signaling and proliferation in CD4^+^ T cells (128), supporting the role of LDL or its major protein component apolipoproteinB100 (apoB100) as autoantigens in atherosclerosis. During disease progression, numbers of circulating and lesional T_regs_ decline and CD4^+^ T effector (T_eff_) populations increase (129). The loss of regulatory T-cells can be attenuated by a switch to a normal diet (129).

At later stages of atherosclerosis, while retaining key features of regulatory T-cells, T_regs_ show mixed, multilineage phenotypes with characteristics of T_H_1 and T_H_17 cells, secrete proinflammatory cytokines, lose their suppressive function and fail to protect from atherosclerosis (89, 96, 129, 130). These data suggest the existence of an LDL-driven immunosuppressive regulatory response in atherosclerosis that undergoes pathogenic conversion during disease progression (Figure 2B). Indeed, studies directly demonstrated the existence of CD4^+^ T-cells with specificity for apoB100-peptides in mice (96) and humans (94, 96), using MHCII multimers loaded with ApoB peptide oligomers (131). In mice, autoreactive T cells specific for ApoB_978−993_ were oligoclonal and expanded in hypercholesterolemic conditions. In early disease, they exhibited a T_reg_-like transcriptome. During disease progression, they converted into T_H_1/T_H_17-like proinflammatory T_eff_ cells that retained only a residual T_reg_ transcriptome. After adoptive transfer, they failed to protect from atherosclerosis, despite their regulatory properties (96). In individuals without atherosclerosis, two thirds of CD4^+^ T cells reactive to another ApoB100 peptide, ApoB_3036−3050_, expressed the T_reg_-defining transcription factor *FoxP3*. In patients with manifest coronary artery disease, this proportion was reduced to 30%, whereas a large proportion showed a mixed, partially proinflammatory T_reg_/T_eff_ phenotype (94). Loss and pathogenic conversion of a suppressive, stabilizing T cell population may tilt the delicate balance of pro- and anti-inflammatory immunity in the plaque and could thus be a key factor driving plaque progression and instability. Preventing the proinflammatory switch of T_regs_ or mobilizing T_reg_ cells to maintain or restore immune tolerance in atherosclerosis bears interesting therapeutic potential. Several promising tolerogenic vaccination approaches, largely focused on ApoB, LDL or oxLDL, are currently being explored (132).

### Clonal Hematopoiesis

**Figure 3:**
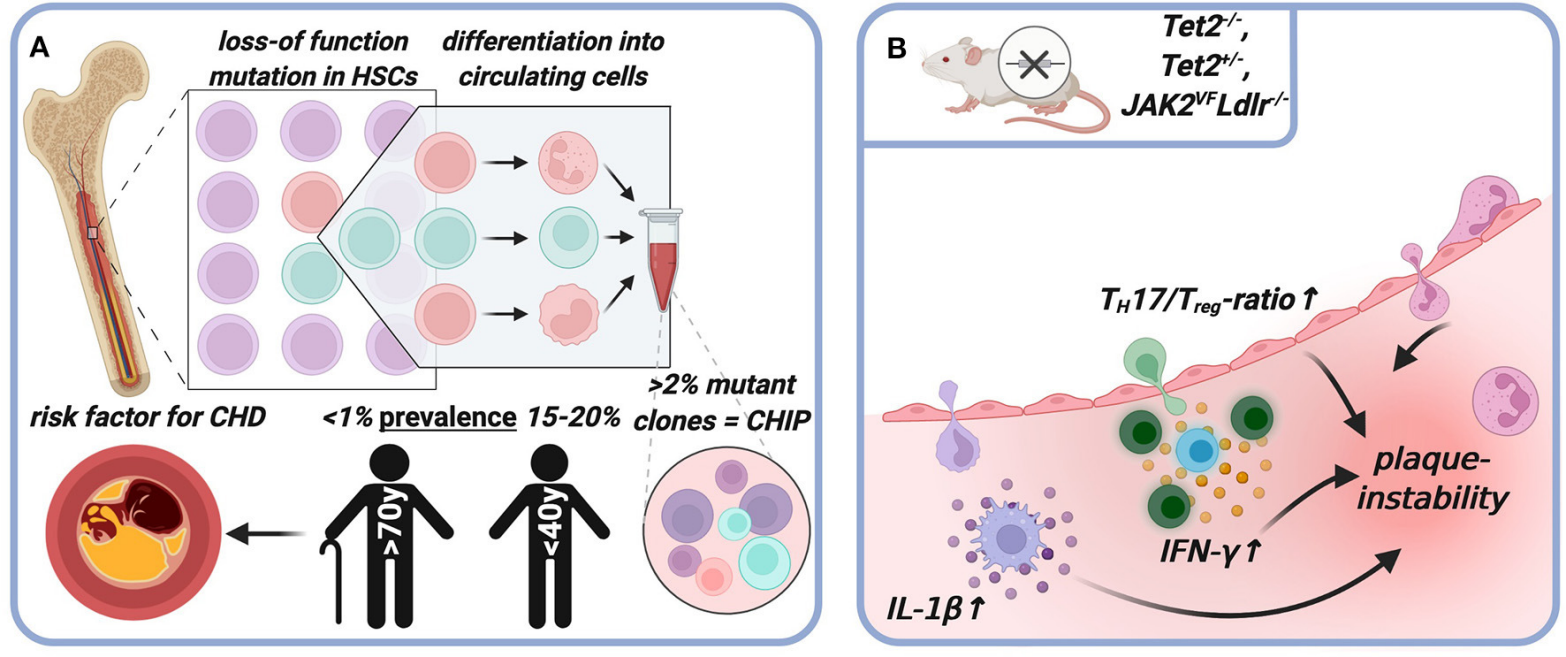
Clonal hematopoiesis and atherosclerotic plaque progression. Sustained somatic mutations in bone marrow hematopoietic stem cells (HSCs) drive their clonal expansion and differentiation into circulating mutated leukocyte clones with myeloid bias **(A)**. Mutated leukocytes invade the plaque and overproduce inflammatory mediators, mutated regulatory T-cells are phenotypically unstable and switch toward inflammatory effector cells **(B)**. Created with BioRender.com.

Sustained somatic mutations in bone marrow hematopoietic stem cells (HSCs), frequently loss-of function mutations of epigenetic transcriptional proliferation regulators commonly associated with myeloid cancers (*DNMT3A, TET2, ASXL1, JAK2*) (133) may impart selective advantages to HSCs and drive their clonal expansion (134, 135). Mutated clones differentiate into circulating granulocytes, monocytes, and lymphocytes (136–138) (Figure 3A). Witness that this is not typically accompanied by blood leukocytosis (137, 139).

If mutant clones are present in ≥2% of peripheral blood leukocytes (variant allele frequency, VAF) and no hematologic malignancy has been diagnosed, this is called clonal hematopoiesis of indeterminate potential (CHIP) (135). Of note, CHIP may occur in the absence of known driver mutations (140, 141) and if present, >90% CHIP carriers harbor only one mutation (141). CHIP-prevalence is strongly age-related, ranging between <1% in those younger than 40 to 15–20% in those older than 70 years (142), which could in part explain the age-related increase in CVD (141). CHIP associates with a 40% increase of all-cause mortality (134) and doubles the risk of incident coronary heart disease (CHD), upstream and independent to traditional risk factors (134, 137). The risk of early-onset MI (<50 years old) quadruples in CHIP carriers as compared to non-carriers (138). If the VAF is 10% or more, CHIP-bearers without symptomatic CHD have dramatically higher coronary calcium scores, a radiological surrogate marker of coronary plaque burden (138).

In the light of these findings, CHIP has emerged as independent risk factor for plaque instability, likely in part due to effects of driver mutations on immune effector cells (141). For some mutations, mouse and human studies suggest causality (Figure 3B): monocytes from patients with DNMT3A mutation showed increased inflammatory gene expression, including those of the NLRP3 to IL-1β to IL-6 to CRP pathway (143). *Tet2*^−/−^ or *Tet2*^+/−^ (138, 139) -as well as *JAK2*^*VF*^*Ldlr*^−/−^ (144) mice had larger atherosclerotic lesions and this was still true when Tet2 was selectively knocked out in the myeloid compartment (138, 139). *Tet2*^−/−^ macrophages accumulated more in the arterial wall (139), and *Tet2*^−/−^ and *JAK2*^*VF*^macrophages had a more proinflammatory phenotype and produced more IL-1β (139, 144). Neutrophils adhered and transmigrated more into larger *JAK2*^*VF*^*Ldlr*^−/−^ lesions and after 12 weeks of HFD plaques displayed signs of instability such as larger NCs (140). Although CHIP driver mutations induce myeloid bias (145), the destabilizing effect appears to extend to other immune compartments: T-cells from patients with *DNMT3A* mutations exhibit highly-inflamed transcriptomes and altered T_H−_signatures (143). Patients with *DNMT3A* mutation showed an increased T_H_17/T_reg_ ratio (146) and increased IFN-y production in T-cells (147). Specific LysM^Cre^-driven deletion of *Tet2* in T_regs_ led to deranged suppressor function, phenotypic dysregulation and eventually to a phenotypic switch toward a proinflammatory T_H_17/T_FH_ effector phenotype (148), similar to what has been described for pathogenic ApoB-reactive autoimmunity in atherosclerosis (96).

Note the recent twist introduced by a new study, which suggests reverse atherosclerosis-CHIP causality (149, 150). HSCs proliferate more in the presence of atherosclerosis, likely due to enhanced inflammation and hypercholesterolemia, which accelerates somatic evolution and increases the risk of expansion of clones that carry CHIP driver mutations. It was demonstrated through a novel mathematical modeling approach that this increase in HSC proliferation alone would suffice to explain increased CHIP prevalence in CVD (149, 150). Taken together, accumulating evidence points to altered inflammatory signaling from expanded leukocyte clones that carry somatic mutations as key driver of plaque instability. Future experimentation will need to strengthen evidence of how CVD and clonal hematopoiesis are linked.

## Plaque Disruption

### Immune Mechanisms of Plaque Rupture

Excessive accumulation of leukocytes, a highly proinflammatory cytokine milieu, necrotic core (NC)-enlargement by cell death, and protease-driven destruction of extracellular matrix (ECM) that results in cap-thinning are hallmarks of unstable plaque (151). Some of these characteristics are likely promoted by the mechanisms discussed above: LDL-trained “memory”-macrophages over-produce TNF-α, IL-6, and Matrix metalloproteinases (MMPs) (23). ApoB-specific proinflammatory T_H_1/T_reg_ and T_H_17/T_reg_ cells that have lost their regulatory, immune-balancing properties (96) exacerbate plaque inflammation, likely mainly by IFN-γ production. The pathogenic proinflammatory switch of T-cells (148) as well as the production of inflammatory cytokines could be accelerated by a disproportionate influx of leukocyte clones that carry a CHIP-driver mutation (143, 146).

Moreover, activated plaque T-cells overexpress the co-stimulatory molecule CD40-ligand (CD40L) (152). Enhanced CD40L gene expression and other proinflammatory cues such as IFN-γ, TNF-α, and IL-1 upregulate its major ligand CD40 on lesional APCs and smooth muscle cells (SMCs) (152). Plaques from hypercholesteremic atherosclerotic mice treated with an anti-CD40L antibody are more matrix-rich and more stable and contain less macrophages and T-cells (153–155), mechanistically explained through decreased VCAM-1 expression (154) and enhanced TGF-β signaling (155); specifically, the binding of T-cell-expressed CD40L to surface-CD40 in DCs enhanced T_H_1 polarization and increased T_H_1-associated IFN-γ production (156). In a CD4^+^ T-cell-specific conditional CD40L-KO model, plaques were less proinflammatory and had smaller NCs and thicker caps (156). Monocyte-to-macrophage differentiation, T-cell-CD40L interaction with macrophage-CD40 (157) and a proinflammatory cytokine-milieu all induce macrophages to overproduce a broad spectrum of highly destructive MMPs (121), which degrade ECM and promote thinning of the fibrous cap. IL-1β-induced chemokines and activated SMCs recruit neutrophils to advanced plaques, where they eject NETs (77, 78), mediators of an act of perfidious targeted killing: the NET-contained nuclear protein cytotoxic histone H4 was recently shown to bind to SMCs and lyse their membrane by pore formation, thereby causing their death (78). SMCs are major producers of elastin, collagen, and other ECM (151). Thus, SMC-death renders the plaque more volatile (158). Plaque stability was rescued by antibody-mediated neutralization H4 or shieling of its functional domain (78). Witness that SMCs are phenotypically highly plastic (Figure 1): In SMCs, cholesterol-uptake induces expression of typical macrophage markers (e.g. CD68 or Mac-2) and foam cell-like features, while SMC contractile genes are downregulated (159). Indeed, within advanced atherosclerotic plaques, several reports identify SMCs, not macrophages, in 30–70% of foam cells (22, 159–163). Using SMC lineage tracing and SMC-specific KO models in ApoE^−/−^ mice, a recent elegant study demonstrated detrimental effects of this SMC-to-macrophage switch on plaque stability (163). Specifically, conditional deletion of the transcription factor Krueppel-like factor 4 (KLF4) prevented SMC switching, but also markedly decreased lesion size and increased fibrous-cap thickness and other indices of plaque stability. Excessive death of SMCs, macrophages and foam cells overcharge the plaque phagocytes' capacities for effective efferocytosis (disposal of dead cells through phagocytosis), leading to failed clearance of dead cells and NC enlargement (164). Caspase-1, activated by NLRP3-inflammasome assembly through, amongst others, intracellular cholesterol crystals (118) causes pyroptosis in the vessel wall, a proinflammatory form of lytic cell death that leads to rapid membrane destabilization and proinflammatory cytokine release (164). Similarly, necroptosis, another highly proinflammatory form of cell death, occurs in advanced plaque (164). Ligation of macrophage-expressed CD40 induces expression of procoagulant tissue factor in the NC (165). When finally, the thinned, weakened fibrous cap ruptures, this prothrombogenic material is exposed to the bloodstream and causes thrombin activation, platelet aggregation and thrombus formation, which leads to vessel occlusion and critical organ ischemia in the heart (35).

### Immune Mechanisms of Plaque Erosion

Despite similar clinical presentation, postmortem studies report substantial histopathological differences between ruptured and eroded plaques that point to distinct underlying pathomechanisms (166). Macrophages, foam cells, and other inflammatory cells are rare in eroded plaques and surface SMCs are relatively abundant (166, 167). Necrotic cores are smaller, calcifications scarce, and extracellular matrix components, especially hyaluronan (HA), predominate (29, 167, 168). Thrombi that overlay erosion are platelet-rich and contain less fibrin and more myeloperoxidase-positive inflammatory cells as compared to thrombi that form on ruptured lesions (27, 28, 169).

Recent comparative studies have increased the granularity of knowledge regarding clinical, morphological and molecular differences between these two ACS-causing entities (29–34). They unanimously corroborate the early notion that the widely studied mechanism of plaque rupture does not pertain to plaque erosion, but that plaque erosion is likely a wholly different disease entity (28, 36). Detailed understanding of the unique pathobiology of plaque erosion is essential. First, the prevalence of plaque erosion is increasing and may, in part due to better control of traditional risk factors, become the dominating plaque morphology in ACS (28, 36, 170). Second, together with targeted immunomodulation, tailored therapy for plaque erosion may clear the path for precision management in ACS. Early pilot studies suggest that non-invasive pharmacological treatment without stenting may be sufficient in plaque erosion (170, 171).

The sparsity of macrophages in eroded plaque and the fact that endothelial denudation is a histological hallmark of plaque erosion (46, 166, 167) primed the early assumption that immune mechanisms and inflammation may not play a central role in the pathophysiology of erosion ACS (167, 168). Recent advances, however, suggest a complex interplay between local mechanical triggers and both an innate and an adaptive immune response as driving forces in plaque erosion.

Eroded plaques preferentially localize near coronary bifurcations (7), and many studies support a key role for turbulent local fluid dynamics as well as consequently altered endothelial shear stress as “first hit” initiators of the cascade that drives plaque erosion (29, 32, 172–175). During atherogenesis, disturbed flow primes lesion-specific overexpression of various toll-like receptors, including toll-like receptor 2 (TLR2) (47, 176), especially in endothelial cells and macrophages. (177–180) Exogenous, but also endogenous ligands such as agonists released during tissue damage or apoptosis, cholesterol crystals or hyaluronic acid, activate TLRs (177–180). Through myeloid differentiation primary response gene 88 (MyD88) and other signaling adapters, TLR-ligation results in activation of interferon response factor (IRF) family members and nuclear factor-kB (NF-kB), leading to expression of leukocyte adhesion molecules and chemoattractants, activation of interferon- and proinflammatory cytokine pathways and generation of matrix metalloproteinases (MMPs). (45, 179, 180) General deficiency of MyD88, TLR2 or TLR4 significantly reduced atherosclerosis and vascular inflammation in mice and humans. (180) At sites of disturbed flow conditions predisposed for plaque erosion (32, 172–175), NF-kB activation through TLR2 has been proposed to facilitate disruption of endothelial cell (EC)-to-extracellular matrix contact, EC apoptosis, and the desquamation of the endothelial monolayer that is characteristic for eroded plaque. (45, 47, 177) Neutrophils adhere to areas of endothelial activation and -denudation, potentiate EC death (45, 47) and release NETs (28), which have been crucially implicated in the pathophysiology of plaque erosion (36, 45–47). NETs sustain a low-level proinflammatory response on the luminal endothelium (36, 77) and promote pathological thrombosis by induction of prothrombotic endothelial tissue factor (181) and by facilitating platelet adhesion, activation, and aggregation (182).

This interplay between endothelial dysfunction, TLR overexpression and neutrophil accumulation and activation is accompanied by a local enrichment of both CD4^+^ and CD8^+^ T-lymphocytes (29). Effector mediators secreted by cytotoxic T cells, such as granzyme A, granulysin, and perforin were shown to further promote EC death and CD8^+^ T-cells, but not monocytes, adhered more to flow-primed endothelium in an integrin-β2 and integrin-α4 dependent manner *in vitro* (29). Adhesive interaction between HA and its major receptor CD44 (183) could provide a link between T-cell recruitment selectively to sites of endothelial erosion: CD44 is overexpressed on antigen-activated T-cells in areas of disturbed flow (184), and HA accumulates in eroded, but not in ruptured plaques (167, 168).

In sum, it seems that after endothelial activation and dysfunction as common initiator, plaque rupture is lipid-driven and kindled by macrophages, while endothelial damage, potentiated by neutrophils, underlies plaque erosion. However, both pathologies critically involve the adaptive arm of the immune system. In this context, interesting questions remain unanswered. If lipids do not play a substantial role in plaque erosion, can lipid lowering prevent this type of ACS? Furthermore, it is known that LDL-derived ApoB-peptides elicit antigen-specific responses and drive T cell activation and formation of memory pools in mouse and human atherosclerosis (94, 96). Since eroded plaques are lipid poor (166, 167), are different antigens involved in plaque erosion? If so, do antigen-specific cells persist in memory pools? And, since helper T-cell subsets may have stabilizing, anti-inflammatory or proinflammatory effects (98), how are helper T-cells differentiated?

## Systemic Influences that Throw Plaques into Crisis

Atherosclerosis is usually a silent, slowly developing, smoldering condition that leads to gradual build-up of plaques in the tunica intima of arteries. In most cases, its first clinical manifestation is a heart attack, which occurs when the plaque abruptly destabilizes, occluding a vessel necessary for the blood supply of the myocardium. Some systemic influences episodically accelerate plaque progression, massively raising the likelihood of destabilization.

### Acute Infections and Cardiovascular Risk—Implications for the COVID-19 Pandemic?

Acute infections abruptly raise the incidence of MI. This risk culminates directly after infection and decreases over time (185–187). Notably, the risk is not pathogen-specific and therefore rather mediated by the host response than by certain elements of microbes. A recent study found a six times higher risk for acute MI during the first 7 days after a diagnosis of influenza A or B and to a smaller extent also for non-influenza respiratory viruses (185). In the very recent IAMI-trial (188, 189) and other smaller trials (190), patients hospitalized for acute MI who were subjected to influenza vaccination had a lower mortality risk and a better cardiovascular outcome than patients treated with a placebo shot. A severe increase in CV-risk was also reported in the context of community-acquired bacterial pneumonia (186). Risk peaked in the first week after infection but was still elevated up to two years after hospitalization (186). Non-respiratory infections like urinary tract infection implicate comparable risk (187). Emerging data point to a similar increase in CV-risk associated with COVID-19-disease (191, 192) and preexisting CVD independently associated with worse outcome in COVID-19 patients (193, 194).

**Figure 4:**
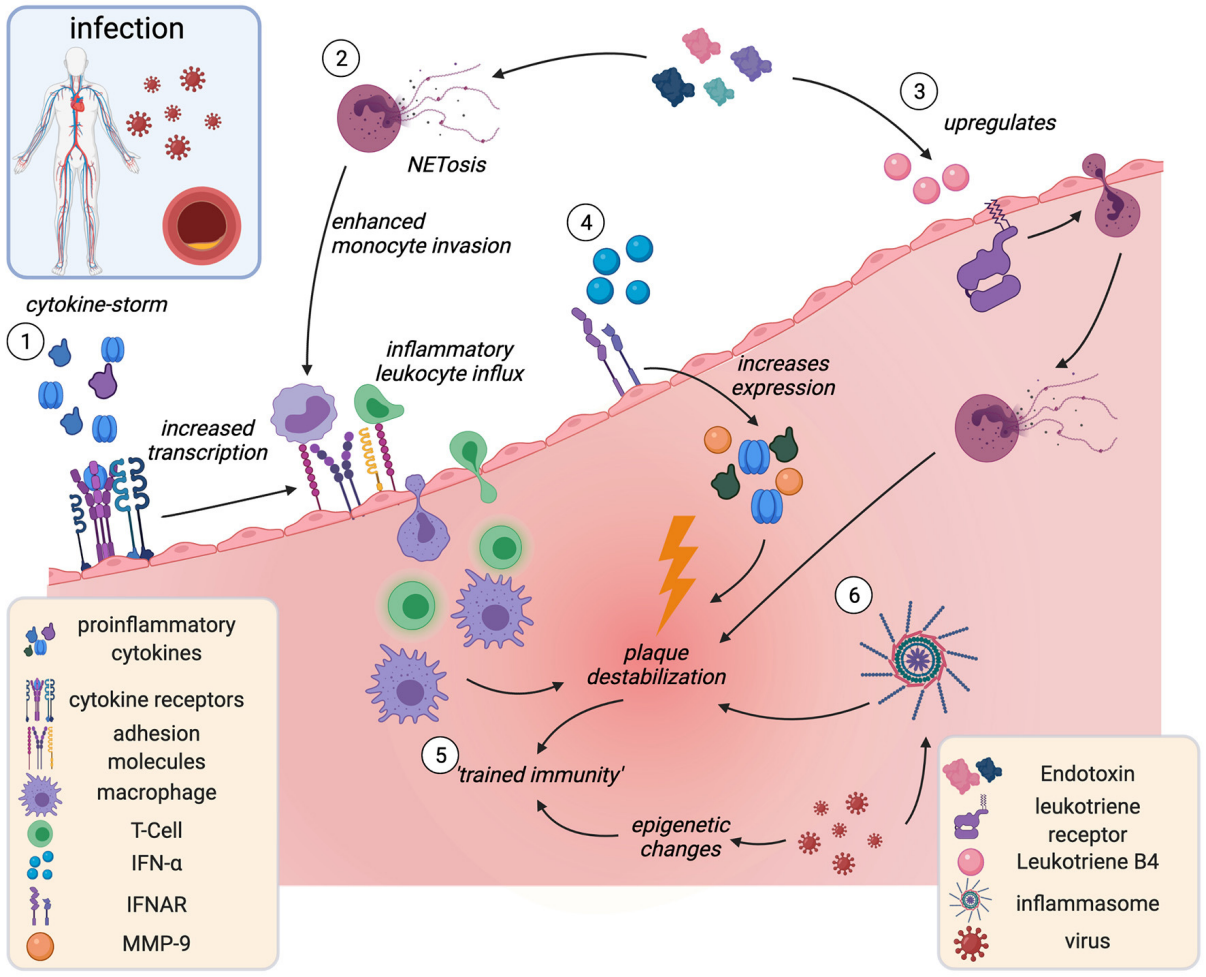
Acute infection and plaque instability. Infection-associated cytokine release induces overexpression of plaque adhesion molecules and subsequent leukocyte influx and accumulation in the plaque (1). Endotoxemia associates with increased intraluminal NETosis (2), thereby increasing monocyte adhesion and Leukotriene B4 release (3). Leukotriene B4 triggers neutrophil invasion and plaque destabilization. IFN-α is released in viral infection and acts as proinflammatory amplifier (4). Infection may act as “training cue” for innate immune memory (trained immunity), leading to hyperreactive, proinflammatory leukocyte phenotypes (5) and inflammasome activation (6). IFN-α, interferon-α; IFNAR, interferon-α receptor. Created with BioRender.com.

Some experimental evidence from mice and humans mechanistically lines this clinical association (Figure 4). In *ApoE*^−/−^ mice infected with influenza A virus, histological analyses showed increased inflammatory macrophage and T-cell accumulation in atherosclerotic plaques (195) and post-mortem analyses showed enrichment in myeloid- and T-cells in the coronary adventitia of patients who died from acute infection (196). Intra-abdominal sepsis accelerated atherosclerosis within only 24 h in *ApoE*-KO mice, in part mediated by increased lesional TNF, IL-6 and CCL2 (197). Endotoxemia upregulated the chemoattractant Leukotriene B4 (LTB4) in aortas of *ApoE*^−/−^ mice, which caused neutrophil invasion and subsequent enhanced collagen digestion, necrosis, and rapid destabilization in plaques (198). Enhanced NET-release in the arterial lumen during endotoxemia increased monocyte adhesion and accumulation (199). Exposure to various microorganisms can induce epigenetic changes that mediate trained immunity (123, 124).

As discussed above, those trained cells are proinflammatory, proatherogenic and have destabilizing effects on the plaque (200). Plausibly, trained immunity has been proposed as a mechanistic link for how infections augment CV risk (200). A breach of immune tolerance by molecular mimicry (201) as well as pathogen-induced inflammasome activation (202) in myeloid cells have been shown to contribute to accelerated atherosclerosis in the context of viral infection.

Although evidence is still scarce, some of the mechanisms by which infections cause plaque vulnerability could pertain to COVID-19: various pathogens upregulate expression of adhesion molecules on ECs, including vascular ECs in mice and humans (203–205). Similarly, COVID-19 patients have elevated plasma levels of leukocyte adhesion molecules VCAM-1, ICAM-1, VAP-1, and PECAM-1 (206, 207). By hyper-acute induction of adhesion molecules in the plaque, systemic infection could enhance inflammatory leukocyte influx and accelerate plaque destabilization (203). Circulating proinflammatory cytokines such as IL-1α, IL-1β, and TNF-α, released in a storm in severe COVID-19 (208), could potentiate this effect and activate lesional inflammatory cells (11). IFN-α, typically released in viral infection (209) and elevated in COVID-19 patients (210), is a proinflammatory amplifier in atherosclerosis plaque (209). It has been shown to increase levels of MMP-9, TNF-α and IL-12, which all contribute to plaque destabilization (209). Accumulating evidence points to endothelial activation and dysfunction as key elements of COVID-19 disease (208, 211). Given this pathophysiological similarity, it could be hypothesized that the COVID-19-associated increase in MI-risk be driven by an increase in the incidence of plaque erosion rather than plaque rupture, but only time will tell if this hypothesis holds.

### Acute Mental Stress—An Increasingly Prevalent Modifiable Risk Factor

**Figure 5:**
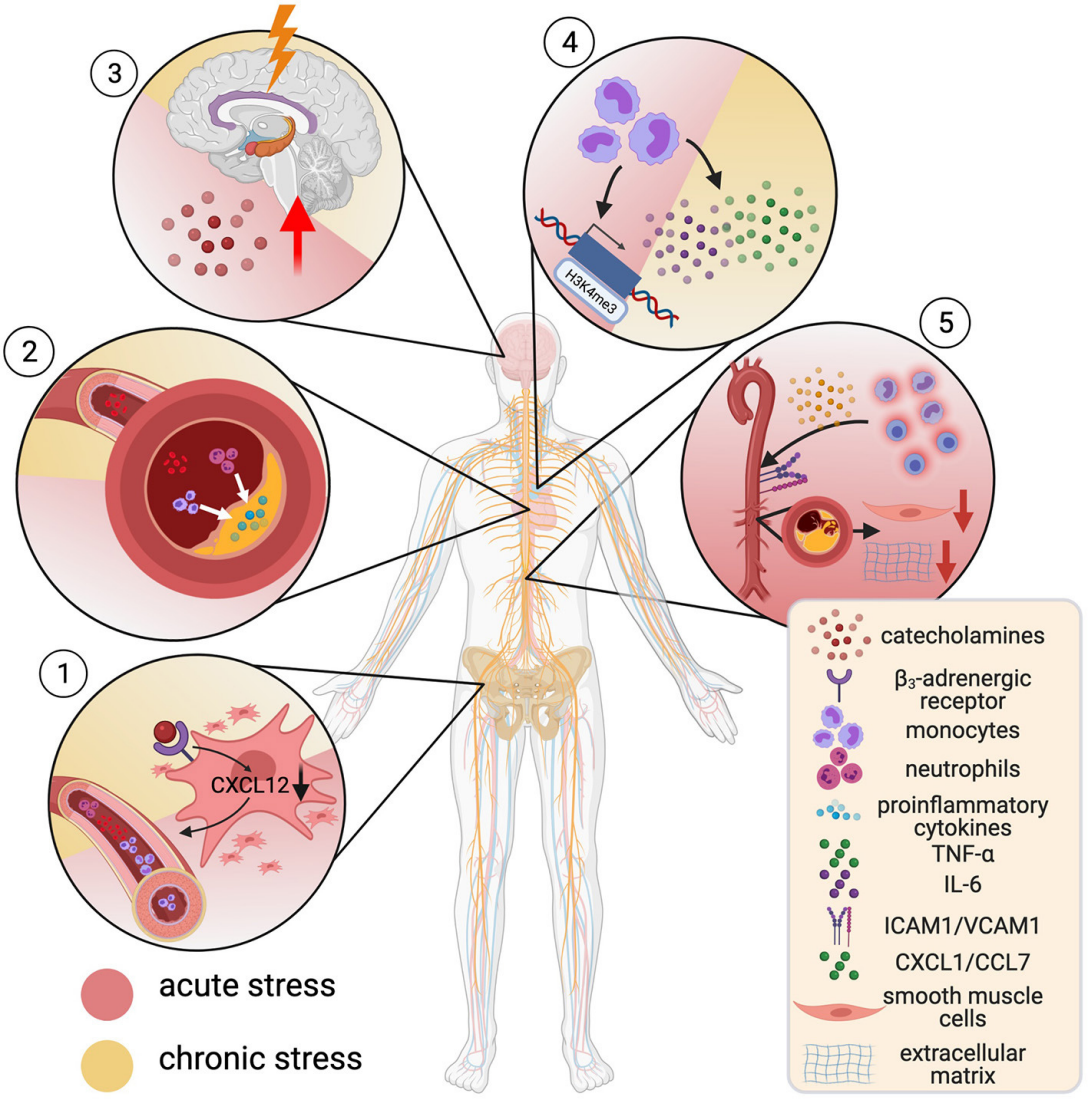
Mental stress and cardiovascular risk. α- and β-adrenergic receptors are expressed on hematopoietic stromal cells and most leukocytes. Catecholamine-binding to β_3_-receptors on stromal cells decreases CXCL12, which increases hematopoiesis, causing blood monocytosis and neutrophilia (1). This associated with accumulation of inflammatory cells in atherosclerotic lesions (2) and creation of a proinflammatory microenvironment. Stress leads to a rise in circulating catecholamines by activation of the limbic system and thus potentiates these effects (3). Noradrenaline-primed monocytes show increased tumor necrosis factor—α (TNF-α) and interleukin-6 (IL-6) production. This hyperresponsive phenotype goes in hand with H3K4me3-enrichment at promotor regions for proinflammatory genes (trained immunity) (4). Acute stress leads to rapid recruitment of inflammatory monocytes and lymphocytes into tissue by enhanced expression of adhesion molecules (ICAM-1, VCAM-1). This promotes a decrease in numbers of smooth muscle cells and breakdown of extracellular matrix (5). Created with BioRender.com.

The incidence of sudden cardiac death and acute MI shot up after the Northridge earthquake in California (212) and the Great East Japan Earthquake (213) as well as during the first days of the Gulf war in Israel (214). Even everyday life acute stressors such as watching the national team play during the World Soccer Championship triggered a steep rise in ACS events (215). Those and other studies established acute stress as independent risk factor for CHD and MI (216, 217), in some studies as strong as hypertension and diabetes (218). Chronic stress exposure as experienced through job loss (219) or marital strain (220) increases the risk for CVD by 40–50% (218) and MI is more common in individuals who experience repetitive or permanent stress (216). Recent studies have characterized a “stress-sensitive neuro-immune axis” (221) between the sympathoadrenal system and the bone marrow that links stress and atherosclerotic plaque instability (Figure 5): stress activates the amygdala and hypothalamus (222), triggering a transient rise in circulating catecholamines through the sympathetic nervous system. Their corresponding α- and β-adrenergic receptors are expressed on hematopoietic stromal cells and most leukocytes (221). Experimentally, surplus noradrenaline released during mild chronic stress triggered β3-adrenergic receptor-mediated decrease of the cytokine CXCL12 in stromal cells, which curtails excess hematopoiesis and mediates HSC quiescence. This accelerated hematopoiesis and, hence, promoted inflammatory blood monocytosis and neutrophilia (223). In *ApoE*^−/−^ mice, blood leukocytosis through chronic stress-related acceleration of hematopoiesis led to inflammatory myeloid cell- and neutrophil accumulation as well as a more inflammatory cytokine profile in atherosclerotic lesions and promoted plaque instability (223). In line, blood leukocytosis was present in humans exposed to chronic stress (221).

Another plausible link between stress, inflammation, and accelerated atherosclerosis was interrogated by a recent study: catecholamine-induced trained immunity. (Nor)adrenalin-primed human monocytes showed a heightened TNF-α and IL-6 production in response to proinflammatory restimulation and upregulation of glycolysis and oxidative phosphorylation (224). Similarly, a hyperresponsive and proinflammatory phenotype was observed in monocytes of patients with chronic catecholamine-overstimulation due to pheochromocytoma, which persisted for 4 weeks after removal of the tumor. Epigenetic profiling of these cells showed a trend toward H3K4me3 enrichment at the promoter regions of proinflammatory genes (224).

A different study provides insight into how the immune response to acute and chronic stress differs. The researchers show that in mice and humans, *acute* mental stress (a key soccer game for humans, immobilization, or predator-prey stress through fox odor in mice) does not cause accumulation, but instead leads to rapid transient depletion of inflammatory monocytes and lymphocytes, although not neutrophils from the peripheral blood. Cell tracing experiments revealed recruitment of these cells to distinct tissues, including atherosclerotic aortas in *ApoE*^−/−^ mice. This was driven by a noradrenaline-mediated upregulation of ICAM1 and VCAM1 on vascular endothelial cells (VECs) and increased levels of the chemoattractants CXCL1 and CCL7, released predominantly by VECs and macrophages. Besides intimal inflammatory myeloid accumulation, plaques showed decreased SMC numbers, more extracellular matrix breakdown and more likely ruptured under stress in a rupture-prone *ApoE*^−/−^ pressure overload-model (218). In a multisystem 18F-fluorodeoxyglucose (FdG) positron-emission tomography (18F-FDG PET) imaging approach, evidence of a correlation of amygdala activation with enhanced bone marrow signal was shown, which associated with worse cardiovascular outcome in patients over a 3.7-year follow-up period (222). Building on this, multisystem 18F-FDG-PET imaging was employed to show concordant vascular inflammation, amygdala-, and bone marrow activation after acute MI, which concurrently returned to baseline signal after 6 months (225).

## Gut Microbiota-Dependent Modulation of Atherogenesis and Plaque Phenotype

Growing evidence suggests a link between the gut microbiome and the pathogenesis and progression of atherosclerosis (226) and plaque vulnerability (227). This link is based on changes in microbial diversity, compositions, and metabolism and intestinal barrier function affecting host physiology at the system level. Additionally, bacterial DNA has also been identified locally in coronary atherosclerotic plaques pointing to the presence of bacteria in atherosclerotic lesions (228), although their causal role for plaque development and vulnerability still remains a matter of debate (229). In this section we will focus on distinct gut bacterial signatures and microbial-related bioactive metabolites that have been increasingly recognized to shape atherosclerotic disease progression and plaque morphology: TMAO (230) and SCFA (231).

### Contributory Role of Gut Dysbiosis in Atherosclerotic Plaque Development and Vulnerability

The human gut microbiota is mainly composed of the phyla *Bacteroidetes, Firmicutes, Actinobacteria, Proteobacteria*, and *Verrucomicrobia, Firmicutes* and *Bacteroides*, although the large majority of the bacteria are members of the phyla *Firmicutes* and *Bacteroides* (232). Alteration of the microbial homeostasis into a so-called dysbiotic state critically affects human health (233), and may contribute to the progression of atherosclerotic vascular disease (230). Importantly, the contributory role of gut dysbiosis for atherosclerotic plaque development should not be confused with earlier studies postulating an atherogenic role of specific microbial pathogens such as *Chlamydia pneumoniae* and *Helicobacter pylori* by directly invading vascular cells and leukocytes and promote inflammatory processes within the vascular wall, which later could not be confirmed in clinical intervention studies (234). Within the gut microbiome, a negative correlation of gut bacterial diversity and plaque size has been observed in a mouse model of high fat diet-induced atherosclerosis (235). Moreover, increased abundance of *Firmicutes* along with an increase in bacterial lipopolysaccharides (LPS) causing endotoxemia has been associated with pro-atherogenic macrophage M1 activation in obesity (236). Importantly, beyond pro-atherosclerotic clinical conditions such as obesity and metabolic syndrome, antibiotic treatment may also lead to disturbance of microbial homeostatis and, thus, critically affect human health. In a recent study, it was shown that administration of a macrolide antibiotic causes microbial alterations characterized by loss of microbial diversity and higher *Firmicutes/Bacteriodetes* ratio (237). Consequently, enhanced CD68-expressing foam cells and increased M1 polarization was found in the plaque content of atherosclerosis-prone ApoE^−/−^ mice. These findings suggest a pathway in which antibiotic administration impacts the inflammatory process within atherosclerotic plaques by affecting the composition and activity of the microbiome.

On the genus level, analysis of fecal samples from healthy individuals and patients with symptomatic atherosclerosis by means of shotgun sequencing has identified the genus *Collinsella* to be enriched in patients with symptomatic atherosclerosis, whereas the abundance of *Eubacterium* and *Roseburia* was higher in healthy controls (238).

This bacterial profile was associated with an altered metagenome marked by enriched genes in the peptidoglycan pathway in symptomatic patients. Notably, peptidoglycan is known to bind to CD14 on macrophages and induce proinflammatory cytokine production (e.g. IL-1, IL-6 and TNF-α) and metalloproteinase 9 in a TLR-2 dependent manner in atherosclerotic plaques and, thus, promotes a vulnerable plaque phenotype (239).

### The Gut Microbial-Host TMA/TMAO Axis: Novel Driver of Atherosclerosis

Beyond changes in the composition of gut microbiota, its metabolic property has been increasingly acknowledged as a potential factor to contribute to atherosclerosis and to shape plaque development. One of the gut microbiota-dependent metabolites that has raised significant attention in recent years is trimethylamine-N-oxide (TMAO), the hepatic oxidation product of the microbial metabolite trimethylamine (TMA) (226, 230). TMA is generated by microbial metabolism of dietary nutrients containing a TMA moiety, such as choline and L-carnitine. Following absorption by the host, TMA is oxidized in the liver by flavin monooxygenase into TMAO and then released into the circulation (226).

This microbial-host TMA/TMAO axis is considered to mediate proatherogenic actions, in particular by promoting endothelial cell dysfunction (240) and macrophage foam cell formation (230). We recently demonstrated that a choline-rich diet increases the differentiation of pro-inflammatory Ly6c^high^ monocytes (241). Accordingly, we found a link between increased TMAO levels and high proportion of intermediate CD14^++^CD16^+^ monocytes subsets in high-risk patients.

Notably, in two clinical studies using optical coherence tomography to assess coronary plaque characteristics, increased plasma trimethylamine-N-oxide levels were linked to increased incidence of plaque rupture supporting a critical role of TMAO in vascular inflammation (227, 242). Beyond the pathomechanistic link between TMAO and atherosclerotic plaque development, TMAO has also been reported to promote atherothrombosis by increased platelet activation (243) and tissue factor expression in endothelial cells (244). In recent studies, microbial enzymes that process the generation of TMA have been targeted using small molecule inhibitors that can modulate TMA/TMAO levels and detrimental cardiovascular effects in experimental settings (245). These studies may open new avenues to develop novel strategies for atheroprotection by targeting specific gut microbial enzymes to modulate their metabolic activity.

### Therapeutic Potential of Dietary Interventions to Modulate Short Chain Fatty Acids

Beyond metabolites with detrimental effects on the cardiovascular system, recent studies have placed a spotlight on microbially produced short chain fatty acids (SCFA), such as acetate, butyrate and propionate, from fiber-rich diet as potentially health promoting metabolites. Dietary intervention studies have highlighted the rapid increase in the levels of bacteria that metabolize dietary plant polysaccharides to produce SCFA including *Roseburia, Eubacterium rectale* and *Ruminococcus bromii* (246). In one of the largest metagenome-wide association studies to date, Jie et al. found markedly reduced butyrate-producing bacteria in patients with atherosclerotic cardiovascular disease as compared to healthy controls (247) highlighting the potential atheroprotective role of butyrate and possibly other short-chain fatty acids.

Subsequent experimental studies have demonstrated that colonizing germ-free *Apoe*^−/−^ mice with butyrate *Roseburia intestinalis* mediates atheroprotecting effects by improving intestinal barrier function and reducing the amount of endotoxin in the bloodstream with subsequent reduction in mRNA levels of *Tnf-*α and *Vcam1* in the aortic wall. Notably, germ-free *Apoe*^−/−^ mice colonized with the *R. intestinalis* showed reduced atherosclerotic lesion size with lower number of macrophages and increased levels of collagen, suggesting that colonization with *R. intestinalis* promotes the stability of atherosclerotic plaques (231).

Another SCFA with potential atheroprotective properties is propionate which has been studied in different models of atherosclerosis. Bartolomaeus et al. studied propionate in an hypertension induced atherosclerosis model by infusion of ApoE^−/−^ mice with angiotensin II (Ang II) (248). While Ang II increased splenic effector memory T-cells (T_EM_) and decreased splenic CD4^+^ naive T-cells (T_N_), these shifts in T-cell populations were prevented upon treatment with propionate. This was accompanied by reduced aortic CD4^+^, CD8^+^ T cell, and F4/80^+^ macrophage numbers after propionate treatment. Similar to the effects on splenic immune cells, propionate decreased the frequencies of aortic CD4^+^ T_EM_ and increased the frequencies of CD4^+^ T_N_ cells. These effects translated into a reduction in atherosclerotic lesion burden. In addition to the systemic effects on T-cell immunity, our group very recently identified alteration of intestinal immune system with beneficial effects on lipid metabolism in an hypercholesterolemic atherosclerotic model (249). In particular, we found an increase of intestinal CD25^+^Foxp3^+^T_regs_ and elevated IL-10 levels in the intestinal microenvironment, which in turn downregulated the expression of the intestinal cholesterol transporter Niemann-Pick C1-like 1 (NPC1L1) with subsequent decrease in intestinal cholesterol absorption. Consequently, propionate reduced pro-atherogenic lipoproteins resulting in reduced atherosclerotic plaque lesions.

Collectively, these findings suggest a significant potential of increasing SCFA bioavailability to prevent and treat atherosclerotic cardiovascular disease. However, their long-term metabolic effects and consequences on cardiovascular outcome need to be further explored in future studies.

## Conclusion

Immune cells orchestrate a chronic smoldering inflammation in the vessel wall that drives atherosclerosis development, progression, and destabilization. Pathogenic conversion of autoreactive regulatory T-cells and smooth muscle cells disrupts the sensitive balance between proinflammatory and protective immunity in the plaque. Leukocyte clones that carry CHIP driver mutations and trained hyperreactive myeloid cells boost this inflammatory response. Systemic infection and acute mental stress escalate plaque inflammation partially by hyper-acute recruitment of proinflammatory leukocytes into the vessel wall. Inflammation likely provides the missing element responsible for the cardiovascular risk that remains despite aggressive lipid-lowering and traditional risk factor control. Recent investigations have demonstrated the potential of anti-inflammatory drugs to lower secondary adverse cardiovascular events, and this is certainly only the beginning. Advances in multi-omics, cell tracing, and machine learning have elucidated a plethora of new possible targets for anti-inflammatory therapy that await trial. Inflammation drives plaque disruption which dramatically manifests as myocardial infarction. This can be caused by rupture of the fibrous cap or superficial erosion, which involves thrombus formation on an intact fibrous cap. The pathomechanisms that underly these two entities seem to be fundamentally different and may require tailored personalized therapeutic strategies. If this holds true in large scale clinical studies, it would implicate a fundamental shift in the management of ACS patients. The discovery of a reliable biomarker of plaque phenotype would entail the possibility that more than one third of ACS patients may not require invasive diagnostics, saving them any associated complications. Our joint future effort should be directed toward developing therapeutic concepts that individually stratify ACS patients according to plaque morphology and inflammatory profile, thus precisely targeting individual needs.

## Author Contributions

TG designed and structured the article and determined contents. TG and AH drafted the first version of the manuscript. TG and PS prepared the figures. DL provided critical revisions for important intellectual content of this article. All authors have read and given final approval of the current version of the article.

## Funding

TG received funding support by grants from the German Cardiac Society (DGK) and the German Centre for Cardiovascular Research (DZHK). PS was supported by the DGK Otto-Hess-Fellowship. DL reports research grants from the DZHK and the BIH.

## Conflict of Interest

DL reports conflict of interest by research and educational grants as well as speaker fees from Abbott Vascular. The remaining authors declare that the research was conducted in the absence of any commercial or financial relationships that could be construed as a potential conflict of interest.

## Publisher's Note

All claims expressed in this article are solely those of the authors and do not necessarily represent those of their affiliated organizations, or those of the publisher, the editors and the reviewers. Any product that may be evaluated in this article, or claim that may be made by its manufacturer, is not guaranteed or endorsed by the publisher.
